# Bacteriological and histopathological findings in cetaceans that stranded in the Philippines from 2017 to 2018

**DOI:** 10.1371/journal.pone.0243691

**Published:** 2021-11-11

**Authors:** Marie Christine M. Obusan, Jamaica Ann A. Caras, Lara Sabrina L. Lumang, Erika Joyce S. Calderon, Ren Mark D. Villanueva, Cristina C. Salibay, Maria Auxilia T. Siringan, Windell L. Rivera, Joseph S. Masangkay, Lemnuel V. Aragones

**Affiliations:** 1 Microbial Ecology of Terrestrial and Aquatic Systems, Institute of Biology, College of Science, University of the Philippines Diliman, Quezon City, Philippines; 2 Natural Sciences Research Institute, College of Science, University of the Philippines Diliman, Quezon City, Philippines; 3 Marine Mammal Research Stranding Laboratory, Institute of Environmental Science and Meteorology, College of Science, University of the Philippines Diliman, Quezon City, Philippines; 4 College of Science and Computer Studies, De La Salle University-Dasmariñas, City of Dasmariñas Cavite, Philippines; 5 Pathogen-Host-Environment Interactions Research Laboratory, Institute of Biology, College of Science, University of the Philippines Diliman, Quezon City, Philippines; 6 College of Veterinary Medicine, University of the Philippines Los Baños, College, Los Baños, Laguna, Philippines; University of Minnesota, UNITED STATES

## Abstract

The relatively high frequency of marine mammal stranding events in the Philippines provide many research opportunities. A select set of stranders (n = 21) from 2017 to 2018 were sampled for bacteriology and histopathology. Pertinent tissues and bacteria were collected from individuals representing eight cetacean species (i.e. *Feresa attenuata*, *Kogia breviceps*, *Globicephala macrorhynchus*, *Grampus griseus*, *Lagenodelphis hosei*, *Peponocephala electra*, *Stenella attenuata* and *Stenella longirostris*) and were subjected to histopathological examination and antibiotic resistance screening, respectively. The antibiotic resistance profiles of 24 bacteria (belonging to genera *Escherichia*, *Enterobacter*, *Klebsiella*, *Proteus*, and *Shigella*) that were isolated from four cetaceans were determined using 18 antibiotics. All 24 isolates were resistant to at least one antibiotic class, and 79.17% were classified as multiple antibiotic resistant (MAR). The MAR index values of isolates ranged from 0.06 to 0.39 with all the isolates resistant to erythromycin (100%; n = 24) and susceptible to imipenem, doripenem, ciprofloxacin, chloramphenicol, and gentamicin (100%; n = 24). The resistance profiles of these bacteria show the extent of antimicrobial resistance in the marine environment, and may inform medical management decisions during rehabilitation of stranded cetaceans. Due to inadequate gross descriptions and limited data gathered by the responders during the stranding events, the significance of histopathological lesions in association with disease diagnosis in each cetacean stranding or mortality remained inconclusive; however, these histopathological findings may be indicative or contributory to the resulting debility and stress during their strandings. The findings of the study demonstrate the challenges faced by cetacean species in the wild, such as but not limited to, biological pollution through land-sea movement of effluents, fisheries interactions, and anthropogenic activities.

## Introduction

The surveillance of wildlife health is part of an early warning system for detecting the emergence or resurgence of disease threats. In the case of cetacean populations in the Philippines, perhaps the most practical way of investigating their health is through their stranding events. A marine mammal is considered stranded when it runs aground, or in a helpless position such as when it is ill, weak, or simply lost [1]. While the event itself deserves attention, as it is not normal for any marine mammal to strand for no apparent reason, each stranded individual can give information on the abundance, distribution, health, and other ecological characteristics of its free-living counterparts [2], as well as threats faced by its population [3]. It is important that stranding events be responded to as quickly as possible, since some stranded animals may quickly die depending on the size of the animal and extent of human intervention [4].

Biases exist in investigating the factors involved in cetacean strandings; easy-to-detect circumstances such as obvious injuries (especially those intentionally inflicted by humans) are likely to be more reported, whereas the role of diseases or parasites may be underestimated. The capacity to detect the presence of pathogens or parasites of stranded cetaceans depends on resources, such as the presence of a stranding network with the capability to respond to stranding events as well as availability of expertise for conducting necropsy and other protocols for case investigation. Nonetheless, whether or not a pathological condition is the underlying cause of a stranding, stranded animals are good representatives for monitoring wildlife health. Also, while live strandings provide good biological samples for laboratory analyses, a dead or decomposing carcass on the beach is just as useful in providing specimens and other information as demonstrated in previous studies.

The available literature on bacteria that were isolated from marine mammals worldwide support the significance of investigating Gram-negative species and their antibiotic resistance or susceptibility. Antibiotic susceptibility patterns have been described for populations and individuals of Atlantic bottlenose dolphins, Pacific bottlenose dolphins, Risso’s dolphins, California sea lions, beluga whale, sea otters and pinnipeds [5–8]. Strains of zoonotic bacteria resistant to multiple antibiotics used for human and animal treatments were isolated from these animals, and some of those bacteria were recognized by the American Biological Safety Association (ABSA) as human pathogens. Associations between increased prevalence of antibiotic resistant bacteria in marine mammals and proximity to human activities were strongly suggested [5, 7, 9–11]. The antibiotic susceptibility profiles of bacteria isolated from cetaceans found in the Philippines where previously reported, wherein more than half of the bacteria (n = 14) had single or multiple resistances to a selection of antibiotics [12].

On the other hand, histopathological assessments proved to be useful in determining probable causes of death or debility of stranded cetaceans worldwide [13–17]. Tissue lesions help confirm parasitic and bacterial infections, co-morbidities, physical injuries (e.g., brought about by fisheries or human interactions) and bioaccumulation of chemical compounds (e.g., persistent organic pollutants) in cetaceans [16, 18–22]. Histopathological assessment is a practical and informative tool that provides pathological evidence and reinforces the necropsy conducted in dead cetaceans as part of the stranding response.

In this study, swab and tissue samples collected from cetaceans that stranded locally from February 2017-April 2018 were subjected to bacterial isolation (with subsequent antibiotic resistance screening) and histopathological assessment. Data on antibiotic resistant bacteria, parasites, and tissue lesions in cetaceans are valuable in evaluating the factors that may be associated with their local stranding events, observed to have increased in recent years [23, 24]. Of the 29 confirmed species in the country, 28 were reported to have stranded from 2005–2018 [24]. A yearly average of 105 cetacean strandings occurred in the country from 2014 to 2018 [24]; 229 events were recorded by the Philippine Marine Mammal Stranding Network (PMMSN) in collaboration with the Bureau of Fisheries and Aquatic Resources (BFAR) from 2017 (n = 121) to 2018 (n = 108) involving 118 dead and 108 live (n = 3 unknown) stranders.

## Materials and methods

All biological samples were collected in coordination with PMMSN and the Marine Mammal Research and Stranding Laboratory (MMRSL) of the Institute of Environmental Science and Meteorology (IESM), University of the Philippines, Diliman (UPD). The marine mammal stranding response and tissue collection is a nationwide effort which is part of the Memorandum of Agreement (MOA) between PMMSN and BFAR. Laboratory work was done at Microbial Ecology of Terrestrial and Aquatic Systems Laboratory (METAS), Institute of Biology, UPD.

### Sample collection

Cetaceans that stranded in the Philippines from February 2017 to April 2018 were opportunistically sampled for tissues and swabs by veterinarians, prosectors, or biologists who were trained by PMMSN in collaboration with BFAR. Swabs were collected from routine and non-routine sites depending on animal disposition and physical preservation, i.e., based on the expanded version of the Code system established by the Smithsonian Institution’s Marine Mammal Events Program [1]. For routine sites, swab samples were collected from the blowhole and anus of live cetaceans. For blowhole area, swabs were inserted into the hole during a breath, gently moved along the wall, and removed during the next breath in live stranders. Whenever possible, exhaled breath condensate (blow) was collected by lowering a sterile petri dish directly over the blowhole and the dish was swabbed afterwards. Anal swabs were collected by inserting rayon swabs into the anal orifice, and gently swabbing the area. Swab samples were also taken from blowhole and anal areas of freshly dead individuals. Swab samples from non-routine sites (e.g., lesions, organs, and abdominal or thoracic fluid) were also obtained from both live and dead animals especially in relation to suspected infection. Tissues were obtained during necropsy following the procedures of Pugliares et al., 2007 [25]. Stranded cetaceans were characterized in terms of species, sex, age class, stranding type, stranding site, and stranding season. Data gathered from the stranding and necropsy reports were include in the analysis.

### Histopathological assessment

Tissue samples (< 1 cm^3^ each) were preserved in 10% neutral buffered formalin, processed by paraffin-embedded technique, sectioned at 5 μm, and subjected to hematoxylin and eosin (H&E) staining. Tissue sectioning and H&E staining technique were performed at Providence Hospital, Quezon City where tissue sections were stained with hematoxylin in water, dehydrated using a series of increasing concentrations of alcohol, and applied with eosin as a counterstain. Stained specimens were passed through xylol and toluol before mounting [26]. Using light microscopy, stained tissue samples were observed for the following: inflammation; fibrosis; granuloma lesions; edema; presence of cysts; endothelial damage (including endothelial deposits); presence of macrophages; granules; microthrombi formation; and hemorrhage.

### Bacterial isolation and antibiotic resistance screening

Swab samples in transport media (e.g., Amies) were stored at 4°C and were sent to the laboratory within 18–24 h. Swabs were then enriched in Tryptic Soy Broth (TSB) for 18–24 h at 37°C. From the enriched media, inocula were streaked on MacConkey Agar (MCA) plates. Morphologically distinct Gram-negative colonies were sub-cultured and purified. Bacterial smears of pure cultures were Gram-stained according to Brown and Smith (2015) [27]. Gram-negative bacterial isolates were subjected to 16S rRNA gene sequencing-based identification and antibiotic resistance screening.

Pure bacterial isolates were identified using 16S rRNA gene amplification. Bacterial DNA was extracted from the purified isolates using either the GF-1 Bacterial DNA Extraction Kit (Vivantis Technologies) following manufacturer’s instructions, or the Boil Lysis Method following Ahmed and Dablool (2017) [28]. The universal 16S rRNA bacterial gene was amplified from the DNA of isolates through polymerase chain reaction (PCR). The primers used for targeting the 16S rRNA gene were 27F (5’-AGAGTTTGATCCTGGCTCAG-3’) and 1541R (5’-AAGGAGGTGATCCANCCRCA-3’) [29, 30]. The PCR reaction mix consisted of: dNTPs, MgCl2, *Taq* DNA polymerase, DNA template, forward and reverse primers, and nuclease-free water. The thermal cycler conditions were as follows: initial denaturation for 2 min at 95°C, 30 cycles of denaturation for 30 s at 94°C, annealing for 30 s at 55–60°C, extension for 30 s at 72°C, and final extension for 7 min at 72°C. Positive controls (*E*. *coli* ATCC^®^ 25922) and blanks (DNA-free templates) were included. PCR products were subjected to agarose gel electrophoresis (AGE) to detect target DNA band. PCR products were then sent to Macrogen (South Korea) for DNA purification and sequencing. PreGap4 and Gap4 (Staden Package 2.0) were used to obtain the consensus sequences [31]. Sequence homologies were determined using NCBI BLASTn search and further analyses were done using BioEdit [32, 33].

Kirby-Bauer Disk Diffusion Assay [34] was performed to determine the sensitivity of the bacterial isolates to antibiotics (Table 1). These antibiotics were chosen based on (1) inclusion in the priority list of WHO for antibiotic resistance research; (2) known use in agriculture and aquaculture; (3) reported susceptibility profiles of bacteria isolated from marine animals worldwide; (4) use during rehabilitation of stranded marine mammals; and (5) known spectrum activity [5, 35–38]. To ensure that only acquired resistances will be observed, antibiotics to which the bacterial isolates have intrinsic resistances were excluded in the assay. The reactions of the isolates to the antibiotics were described as Susceptible (S), Intermediate (I), or Resistant (R) based on Clinical and Laboratory Standards Institute (CLSI) M31-A2 (2002), M100-S24 (2014), and European Committee on Antimicrobial Susceptibility Testing (EUCAST) v 8.0 (2018). *E*. *coli* ATCC® 25922 was used as the control [39–41]. Multiple Antibiotic Resistance (MAR) Index values were computed using the formula: (# of resistant antibiotics / total # of antibiotics tested) [40]. MAR indices greater than 0.2 were interpreted to come from sources where antibiotics are often used [35, 42, 43]. Also, MAR isolates were interpreted as those that are resistant to three or more antibiotic classes [44].

**Table 1:** Antibiotics used in the Kirby-Bauer Disk Diffusion Assay.

Antibiotic class	Antibiotics
Carbapenems	Imipenem
	Meropenem
	Ertapenem
	Doripenem
Penicillins	Ampicillin
Cephems	Cephalothin
	Ceftriaxone
	Cefoxitin
Fluoroquinolones	Moxifloxacin
	Ciprofloxacin
	Ofloxacin
Aminoglycosides	Amikacin
	Gentamicin
Tetracyclines	Tetracyclines
	Oxytetracyclines
Phenicols	Chloramphenicol
Folate pathway inhibitors	Trimethoprim-sulfamethoxazole
Macrolides	Erythromycin

## Results

**Fig 1:**
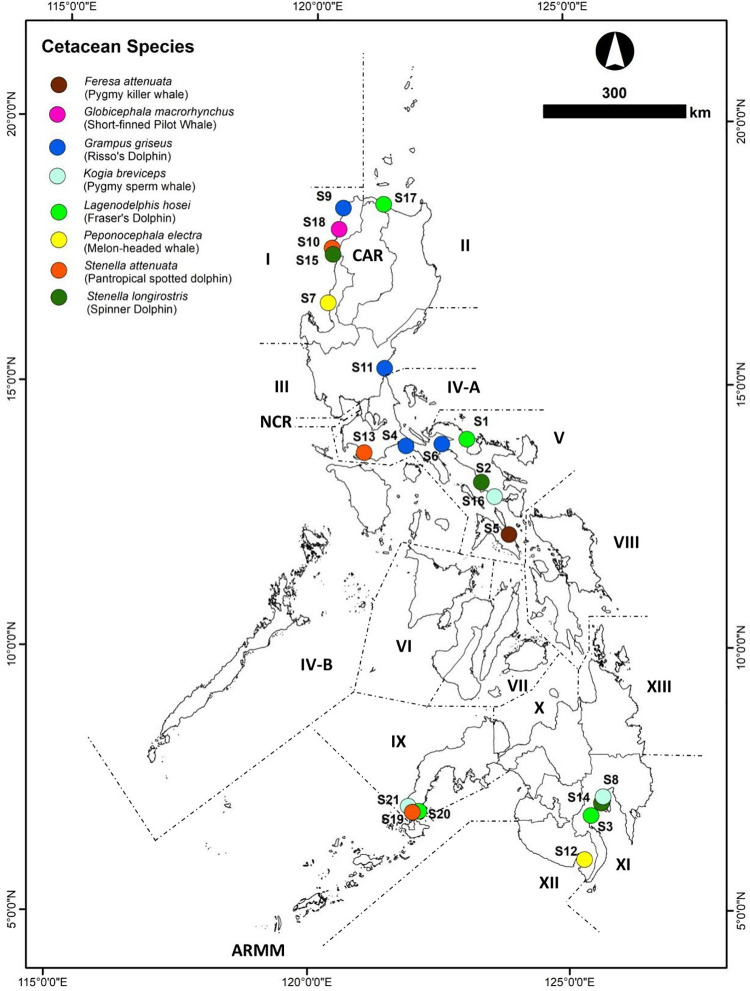
Sites of cetacean stranding events from February 2017-April 2018. S1 –S21: Cetacean Strander Codes; I-XIII, CAR, NCR and ARMM: Administrative Regions in the Philippines (Reprinted from Philippines—Subnational Administrative Boundaries under a CC BY license, with permission from The Humanitarian Data Exchange, original copyright 2020).

In this study, tissue samples and bacterial isolates were obtained from 21 stranded cetaceans representing eight species (*Feresa attenuata*, *Kogia breviceps*, *Globicephala macrorhynchus*, *Grampus griseus*, *Lagenodelphis hosei*, *Peponocephala electra*, *Stenella attenuata*, and *Stenella longirostris*) (Fig 1). Of the 21 select cases sampled, 15 were originally live stranders and six (6) fresh dead. These stranded cetaceans came mainly from Luzon (n = 14) and Mindanao (n = 7). The stranded marine mammals sampled consisted of four (4) Fraser’s, four (4) Risso’s, three (3) spinner, and three (3) pantropical spotted dolphins, and one (1) short-finned pilot, three (3 pygmy sperm, and two (2) melon-headed whales. Samples came from 16 females, four (4) males and two (2) undetermined. By age class, the samples were composed of 15 adults, five (5) subadults, and one (1) neonate (Table 2).

**Table 2:** Stranded cetaceans sampled for the study (2017–2018).

Strander Code	PMMSN Code	Species	Region	Date of Stranding	Stranding Type	Age Class	Condition	Sex
S01	Lh03R5270217	Fraser’s dolphin *Lagenodelphis hosei*	V	27-Feb-17	Single	Adult	Alive	Male
S02	Sl21R5040317	Spinner dolphin *Stenella longirostris*	V	04-Mar-17	Single	Adult	Alive	Female
S03	Lh03R11010317	Fraser’s dolphin *Lagenodelphis hosei*	XI	09-Mar-17	Single	Adult	Dead	Female
S04	Gg04R4A290317	Risso’s dolphin *Grampus griseus*	IV-A	29-Mar-17	Single	Subadult	Alive (Died)	Male
S05	Fa02R5020517	Pygmy killer whale *Feresa attenuata*	V	02-May-17	Mass	Adult	Alive (Died)	Unknown
S06	Gg15R5090517	Risso’s dolphin *Grampus griseus*	V	09-May-17	Single	Adult	Alive (Died)	Unknown
S07	Pe04R1300417	melon-headed whale *Peponocephala electra*	I	30-April-17	Single	Adult	Dead	Female
S08	Kb07R11160517	Pygmy sperm whale *Kogia breviceps*	XI	16-May-17	Single	Adult	Alive	Male
S09	Gg10R1150617	Risso’s dolphin *Grampus griseus*	I	15-Jun-17	Single	Neonate	Alive	Female
S10	Sa18R1210617	pantropical spotted dolphin *Stenella attenuata*	I	21-Jun-17	Single	Subadult	Dead	Female
S11	Gg02R3230617	Risso’s dolphin *Grampus griseus*	III	23-Jun-17	Single	Adult	Dead	Female
S12	Pe06R12030717	melon-headed whale *Peponocephala electra*	XII	03-Jul-17	Single	Adult	Alive	Male
S13	Sa03R4A280717	pantropical spotted dolphin *Stenella attenuata*	IV-A	28-Jul-17	Single	Subadult	Alive	Female
S14	Sl06R11310817	spinner dolphin *Stenella longirostris*	XI	31-Aug-17	Single	Subadult	Alive	Female
S15	Sl23R1300917	spinner dolphin *Stenella longirostris*	I	30-Sep-17	Single	Subadult	Dead	Female
S16	Kb02R5091117	pygmy sperm whale *Kogia breviceps*	V	09-Nov-17	Single	Adult	Alive	Female
S17	Lh04R2011217	Fraser’s dolphin *Lagenodelphis hosei*	II	01-Dec-17	Single	Adult	Alive	Female
S18	Gm11R151217	short-finned pilot whale *Globicephala macrorhynchus*	I	05-Dec-17	Single	Adult	Alive	Female
S19	Sa03R9160118	pantropical spotted dolphin *Stenella attenuata*	IX	16-Jan-18	Single	Adult	Alive	Female
S20	Lh01R9170418	Fraser’s dolphin *Lagenodelphis hosei*	IX	17-Apr-18	Single	Adult	Dead	Female
S21	Kb01R9260418	pygmy sperm whale *Kogia breviceps*	IX	27-Apr-18	Single	Adult	Alive	Female

**Fig 2:**
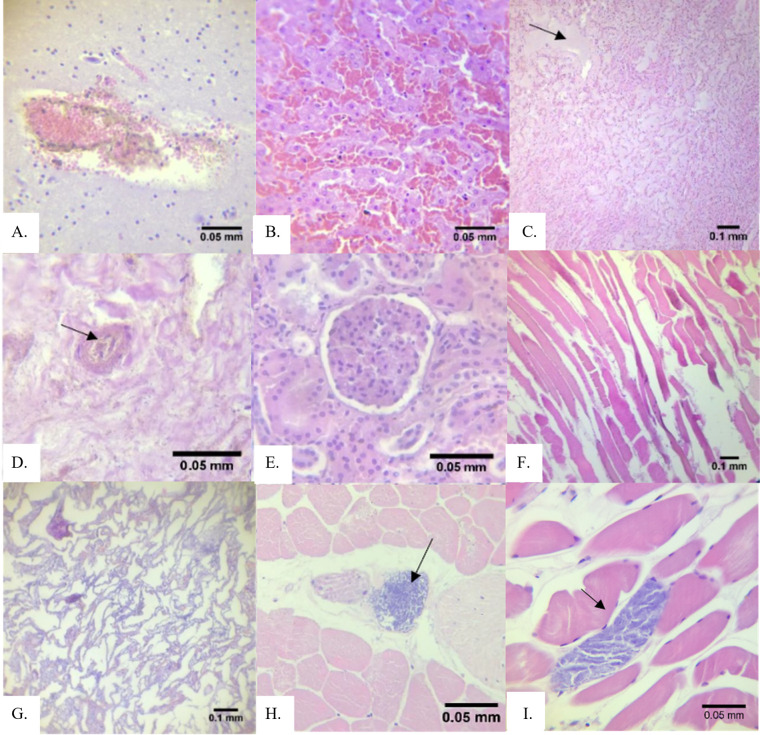
Histopathological lesions observed in tissues of 21 cetaceans that stranded in the Philippines (2017–2018). (A) hemorrhage in S03 kidney; (B) severe congestion in S11 liver; (C) edema in S12 liver; (D) hemosiderosis characterized by the presence of brown granular pigments in S05 kidney; (E) glomerulopathy in S14 kidney; (F) Zenker’s necrosis characterized by hyaline degeneration, loss of striations, and muscle fiber waviness in S06 skeletal muscle; (G) atelectasis (collapsed alveoli) in S10 lungs; (H) unidentified cyst in skeletal muscle of S14; and (I) putative *Sarcocystis* cyst in skeletal muscle of S08.

A total of 73 tissue samples representing 6 organs (brain, cardiac muscle, kidney, skeletal muscle, liver, lungs) were obtained from 21 stranded cetaceans: 3 spotted dolphins (*S*. *attenuata*), 3 spinner dolphins (*S*. *longirostris*), 4 Fraser’s dolphins (*L*. *hosei*), 4 Risso’s dolphins (*G*. *griseus*), 3 pygmy sperm whales (*K*. *breviceps*), 2 melon-headed whales (*P*. *electra*), 1 pygmy killer whale (*F*. *attenuata*) and 1 short-finned pilot whale (*G*. *macrorhynchus*). Of these animals, 19 (90.48%) showed lesions in the organs tissues collected (Table 3 and Fig 2). Most of these cetaceans were adults; there was only one neonate. Unidentified cysts and putative *Sarcocystis* sp. were observed in some tissues with prevalence rates of 47.62% and 9.52% respectively. Some of the unidentified cysts are hypothesized to be other coccidian cysts based on observed structures (e.g., size, shape, thick or thin membrane, etc.) very similar to any stage of reference species (e.g., *Toxoplasma*), however in the absence of confirmatory methods such as immunohistochemical staining, these cysts are labeled as “unidentified”, as observed in H & E stained tissues. Also, *P*. *delphini* cysts in the muscle-blubber region and nematodes in the stomach were seen during gross necropsy and the reported identification was confirmed by the authors.

**Table 3:** Histopathological findings/remarks on cetaceans that stranded in the Philippines from February to April 2018.

Strander No.	Species	Sex	Age Class	Findings
S10^1^	*Stenella attenuata*	Female	Subadult	moderate congestion, hemorrhage, and membranous glomerulopathy in the kidney; unidentified cysts in the skeletal muscle; moderate to severe congestion in the liver; atelectasis in the lungs; no apparent lesion in brain and cardiac muscle
S13	*Stenella attenuata*	Female	Subadult	glomerulopathy and edema in the kidney; no apparent lesion in brain and cardiac muscle
S19	*Stenella attenuata*	Female	Adult	severe congestion in the cardiac muscle; hemorrhage, severe congestion, glomerulopathy in the kidney
S02	*Stenella longirostris*	Female	Adult	moderate congestion in the brain; no apparent lesion in cardiac muscle, kidney, and skeletal muscle
S14	*Stenella longirostris*	Female	Subadult	moderate congestion for cardiac muscle; glomerulopathy with lymphocytic aggregation and unidentified cysts in the kidney; unidentified cyst and *Sarcocystis* cyst in the skeletal muscle
S15^2^	*Stenella longirostris*	Female	Subadult	no apparent lesions in the brain, cardiac muscle, skeletal muscle, liver, and lungs
S01^3^	*Lagenodelphis hosei*	Male	Adult	moderate congestion in the brain; unidentified cyst in the skeletal muscle; no apparent lesions in the cardiac muscle and kidney
S03^4^	*Lagenodelphis hosei*	Female	Adult	moderate congestion in the brain and cardiac muscle; unidentified cysts in the cardiac muscle; severe congestion, hemorrhage, and edema in the kidney; no apparent lesion in skeletal muscle
S17	*Lagenodelphis hosei*	Female	Adult	glomerulopathy and edema in the kidney; unidentified cyst in the skeletal muscle;
S20	*Lagenodelphis hosei*	Female	Adult	severe congestion in the brain, cardiac muscle and kidney; glomerulopathy in the kidney
S04	*Grampus griseus*	Male	Subadult	severe congestion in the cardiac muscle and kidney; no apparent lesion in brain and skeletal muscle
S06	*Grampus griseus*	Unknown	Adult	atrophy and Zenker’s necrosis in the skeletal muscle; no apparent lesion in brain and cardiac muscle
S09	*Grampus griseus*	Female	Neonate	severe congestion in the cardiac muscle; unidentified cyst in the skeletal muscle; severe diffused hepatic sinusoidal congestion in the liver; severe congestion and focal pulmonary edema in the lungs; no apparent lesion in kidney
S11^5^	*Grampus griseus*	Female	Adult	no apparent lesion in cardiac muscle
S07	*Peponocephala electra*	Female	Adult	swollen glomerulus and hemosiderosis in the kidney; unidentified cysts in the skeletal muscle; no apparent lesion in cardiac muscle
S12	*Peponocephala electra*	Male	Adult	membranous glomerulopathy in the kidney; hepatic edema in the liver; pulmonary edema in the lungs; no apparent lesion in brain, cardiac muscle, and skeletal muscle
S08^6^	*Kogia breviceps*	Male	Adult	putative *Sarcocystis* cyst in the skeletal muscle; no apparent lesion in brain and cardiac muscle
S16	*Kogia breviceps*	Female	Adult	severe congestion in the brain; unidentified cyst in the cardiac muscle; hemorrhage and severe congestion in the kidney
S21	*Kogia breviceps*	Female	Adult	moderate congestion in the cardiac muscle; hemorrhage and glomerulopathy in the kidney
S05	*Feresa attenuata*	Unknown	Adult	moderate to severe congestion; hemorrhage and hemosiderosis in the brain; unidentified cysts and hemosiderosis in the kidney; no apparent lesion in skeletal muscle
S18	*Globicephala macrorhynchus*	Female	Adult	moderate congestion in the cardiac muscle; hemorrhage and glomerulopathy in the kidney; unidentified cyst in the skeletal muscle; no apparent lesion in the brain

^1^ acoustic trauma likely cause of stranding.

^2^ only subadult animal without any apparent lesions in organs examined.

^3^ unidentified parasites on eyes and *P*. *delphini* cyst in the skeletal muscle seen during necropsy.

^4^ shark attack likely cause of stranding.

^5^ only adult animal without apparent lesions.

^6^ *P*. *delphini* cysts in the muscle-blubber and nematodes in the stomach seen during necropsy.

Observed tissues include brain, cardiac muscle, kidney, skeletal muscle, liver, and lungs tissues; tissues not mentioned in the findings are those that were not available for histopathological observation.

**Fig 3:**
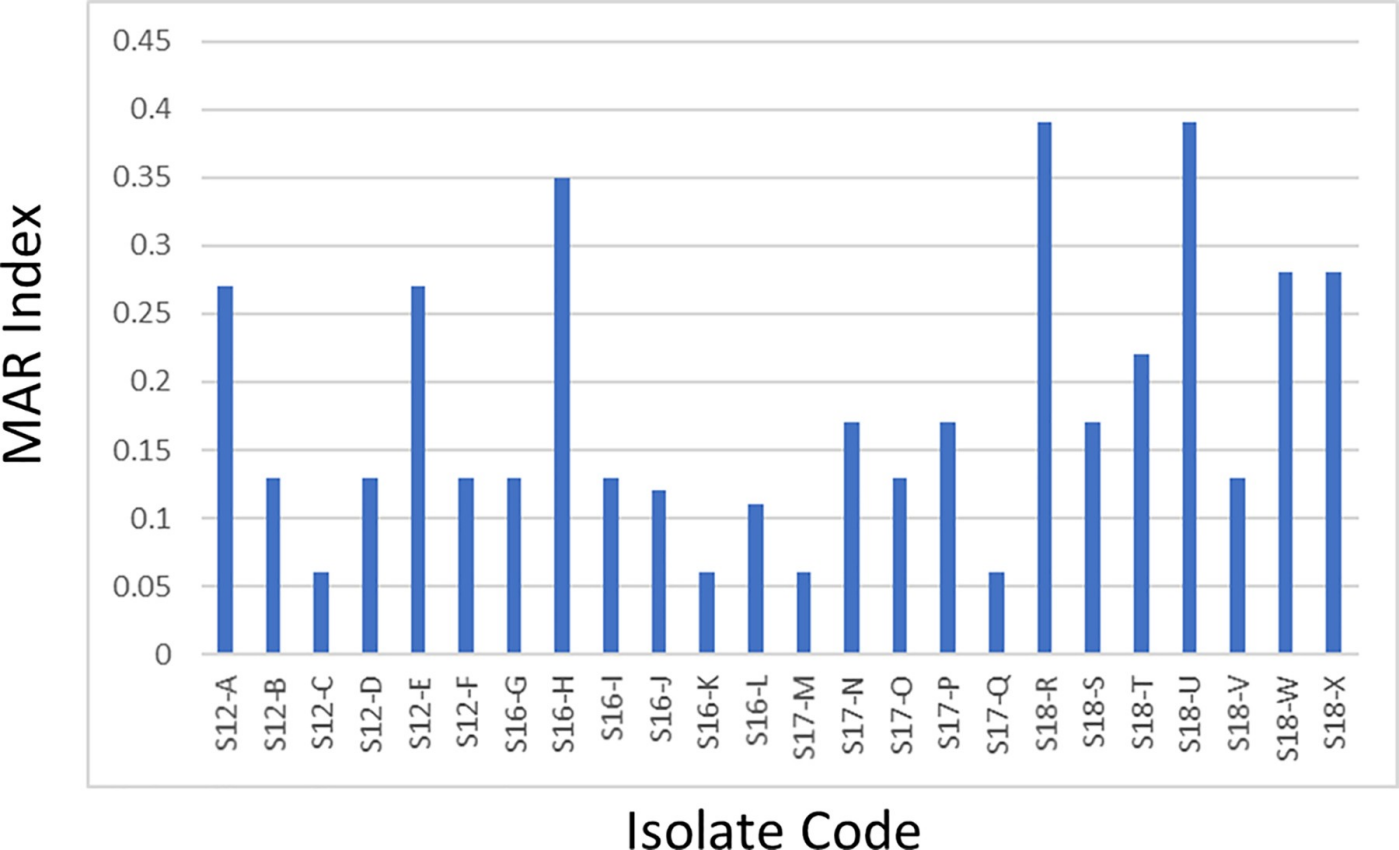
MAR index values of Enterobacteriaceae from sampled cetaceans.

A total of 24 Gram-negative bacteria that belong to the family Enterobacteriaceae were isolated from four cetaceans (S12, S16, S17, S18). Based on 16S rRNA gene, these isolates were confirmed to have 98–100% sequence similarities to species belonging to the following genera: *Escherichia* (n = 6), *Enterobacter* (n = 8), *Klebsiella* (n = 5), *Proteus* (n = 4), and *Shigella* (n = 1) (Table 4). These isolates were resistant to at least one antibiotic class tested, and 79.17% were classified as multiple antibiotic resistant (i.e., resistant to at least three antibiotic classes). The MAR index values of the isolates ranged from 0.06 to 0.39. (Fig 3).

**Table 4:** Genotypic identification of bacteria isolated from stranded cetaceans.

Source Cetacean (Code)	Swab Site	Isolate Code	Nearest Phylogenetic Affiliation (% Sequence Similarity)	NCBI* Accession Number
S12 (*Peponocephala electra*, adult)	urine	S12-A	*Enterobacter cloacae* (99%)	MH101512.1
		S12-B	*Klebsiella aerogenes* (99%)	CP024883.1
		S12-C	*Escherichia hermannii* (98%)	JN644551.1
		S12-D	*Enterobacter* sp. (99%)	KC236445.1
		S12-E	*Enterobacter cloacae* (100%)	KY492312.1
		S12-F	*Enterobacter cloacae* (99%)	JN644583.1
S16 (*Kogia breviceps*, adult)	blowhole	S16-G	*Enterobacter ludwigii* (99%)	JQ659806.1
		S16-H	*Escherichia hermannii* (99%)	JN644551.1
		S16-I	*Enterobacter cloacae* (99%)	KM538690.1
		S16-J	*Klebsiella pneumoniae* (99%)	FO203501.1
		S16-K	*Klebsiella pneumoniae* (99%)	KJ803907.1
		S16-L	*Shigella* sp. (99%)	KU362661.1
S17 (*Lagenodelphis hosei* adult)	genital Slit	S17-M	*Klebsiella pneumoniae* (99%)	CP020847.1
		S17-N	*Escherichia coli* (99%)	AP017620.1
	blowhole	S17-O	*Enterobacter cloacae* (99%)	CP010512.1
		S17-P	*Escherichia coli* (99%)	JQ661149.1
	wound	S17-Q	*Klebsiella quasipneumoniae* (99%)	CP014696.2
S18 (*Globicephala macrorhynchus*, adult)	anus	S17-R	*Proteus mirabilis* (99%)	CP015347.1
	brainstem	S17-S	*Proteus mirabilis* (99%)	CP015347.1
	cerebellum	S17-T	*Proteus mirabilis* (99%)	CP004022.1
	lungs	S18-U	*Escherichia fergusonii* (99%)	KJ803900.1
	blowhole	S18-V	*Enterobacter tabaci* (99%)	NR_146667.2
		S18-W	*Proteus mirabilis* (99%)	CP015347.1
		S18-X	*Escherichia coli* (99%)	CP027060.1

*National Center for Biotechnology Information.

## Discussion

### Lesions in tissues of stranded cetaceans

Histopathological assessment of tissues is a very useful tool to identify factors causing the death of stranded cetaceans or determine the cause of their stranding events [15, 45–47]. However, our histopathological findings were mainly limited to indicative debility and stress during the stranding events because of the inadequate gross descriptions available from the stranding and necropsy reports, prohibiting us to link these findings to disease processes particularly those contributory to cetacean mortalities. As the stranding network (and the country in general) is still building the expertise in performing necropsy for investigating the death of stranded cetaceans, we tried to gather scientific information by performing histopathological observations of available tissues as ancillary to the stranding report. We recognize that there is inadequate information which will help us ascertain the cause of death of the stranded animal, but at the same time deem our findings useful in providing information about the health of the cetaceans.

In general, lesions in tissues of cetaceans were associated with bycatch, trauma, parasitic and bacterial infections, and presence of persistent organic pollutants in stranded cetaceans [13–15, 17, 20, 48–50]. A previous study in the Philippines involved the histopathological assessment of renal tissues which corroborated the results of molecular and culture methods for a suggested case of leptospirosis in a melon-headed whale (*Peponocephala electra*) [51].

When a cetacean strands, it is highly likely to have congestion in the liver and other organs due to the pressure from the weight of its body lying on the thorax as well as immobility preventing venous circulation [52]. It may be noted that organ congestion is the most observed type of lesion in this study, i.e., observed in at least one organ tissue of 13 cetaceans (62%). Several factors can also put cetaceans in stressful situations which can induce stress myopathy and possibly cause congestion and hemorrhage [53]. The stranding event itself can induce trauma and stress myopathy on the animal, causing congestion, hemorrhage, and skeletal and cardiac muscle degeneration such as in the case of Zenker’s necrosis [15, 53, 54] in the skeletal muscle of an adult Risso’s dolphin. However, we cannot corroborate these assumptions with other evidence.

Congestion in brain and kidneys of cetaceans has also been associated with acoustic trauma [55]. One of the ways to confirm acoustic trauma is through histological observations of the inner ears [56]. Acoustic trauma was suggested as the cause of some previously reported cetacean stranding events in the Philippines, possibly due to blast fishing activities near the stranding sites [23, 57]. There is a growing concern on marine environment being compromised by human activities (e.g. underwater explosions, seismic exploration, shipping, operation of naval sonar) which affects the physiology, communication, behavior and energetics of several population of marine species [58–60]. Anthropogenic noise is now recognized as a major global pollutant and is acknowledged as an environmental stressor [58]. Thus, future efforts should include histopathological examinations of the inner ear.

Glomerulopathy was observed in 10 out of 17 cetaceans (59%) with kidney tissues available for observation, and is the most observed kidney tissue lesion (10 out of 14 with lesions or 71%). Comparably, membranous glomerulonephritis was a common finding among stranded cetaceans in Brazil [17]. This lesion was suggested in other studies to be associated with microbial infections or chronic exposure of cetaceans to metals such as cadmium, copper, and zinc, but this remains speculative in our case due to the lack of toxicological analyses and conclusive diagnoses of infections or diseases [50, 61].

Parasites in cetaceans may predispose these animals to bacterial infections, cardiovascular complications, septicemia and other conditions, which are also frequently reported as probable causes of death during their stranding events [16, 62, 63]. Here, we are reporting the detection of cysts in the observed tissues of cetaceans. There is no known histopathological report on cysts such as for example, *T*. *gondii* and *Sarcocystis* sp., in tissues sampled from cetaceans that stranded in different sites in the Philippines, although there are earlier reports on *T*. *gondii* detection using serological and molecular methods [51, 64]. However, as mentioned, we did not perform confirmatory methods for the identification of the cysts, and so we refer to them as either “unidentified” or “putative”. A better understanding of the biology, epidemiology, and pathogenesis of tissue-encysting coccidian organisms that parasitize marine mammals is needed to properly assess the risks and burden of protozoal disease in aquatic ecosystems [65–67]. The transmission of these parasites is still poorly understood in marine mammals, although it is known that they are found in striated muscles of intermediate hosts [68–71]. The most likely modes of transmission of these parasites to aquatic animals are via ingestion of water-borne oocysts or sporocysts originating from sewage runoff or through infected prey [65, 66, 72–74]. During the past two decades, coccidian infections have been detected in marine mammals that stranded along the coast of the northeastern Pacific Ocean [65, 75]. These infections include encephalitis, myositis, hepatitis and myocarditis [66, 67].

In addition, the presence of *P*. *delphini* in the muscles and blubber of stranded Fraser’s dolphin (*L*. *hosei*) and pygmy sperm whale (*K*. *breviceps*) was reported in the necropsy reports. This parasite has been documented in many cetacean species, commonly in the subcutaneous blubber with typical concentration in the perigenital region [76]. Siquier and Le Bas (2003) suggested that Fraser’s dolphins (*Lagenodelphis hosei*) could act as intermediate or accidental hosts for *P*. *delphini*, and that definitive host infection could occur through predation. There is a need for more evidence to confirm the role of cetaceans in the life cycle of this parasite [77]. The consumption of muscles containing these parasites is one of the major routes of transmission to humans. This route of transmission is unlikely to involve cetaceans in the Philippines, as hunting and killing of marine mammals are prohibited under Section 4 of Republic Act 9147 (Wildlife Resources Conservation and Protection Act of the Philippines). Still, there were local reports of fishermen butchering cetaceans for food consumption (pers comm., BFAR Region V).

### Antibiotic resistant bacteria from stranded cetaceans

Overall, the bacterial isolates have resistances to carbapenems and third-generation cephalosporins. Enterobacteriaceae resistant to carbapenems and third-generation cephalosporins are considered a research priority for the discovery of new antibiotic agents [38]. As the “last line of defense” against multiple antibiotic resistant bacteria, the detection of carbapenem-resistant strains is a troubling point of concern as carbapenems are fourth- generation antibiotics recommended for critical Gram-negative infections [78]. To the best knowledge of the authors, only Greig et al. (2007) had so far used imipenem and meropenem for antibiotic susceptibility tests on bacteria isolated from cetaceans. Greig et al. reported imipenem-resistant *E*. *coli* in bottlenose dolphins, but all of their isolates were still susceptible to meropenem at the time [5].

All isolates were most resistant to erythromycin. The high frequency of resistance against this antibiotic is said to be due to acquired macrolide–lincosamide–streptogramin B (MLS) resistance genes, which is common among Enterobacteriaceae [79, 80]. More than 50% of the isolates were also resistant to cephalothin, ampicillin, and moxifloxacin. It must be noted that the isolated *Klebsiella* spp., and *Escherichia* spp., bacterial species often reported as pathogenic to cetaceans, were resistant to erythromycin [81, 82]. Similarly, high resistance to erythromycin, cephalothin, and ampicillin of *E*. *coli* isolated from bottlenose dolphins in Florida and South Carolina was reported [5, 6]. Extra-intestinal pathogenic *E*. *coli* isolated from resident killer whales of San Juan Islands, Washington, were found to be resistant to aminoglycosides, sulfonamides, and tetracycline [83]. Resistances against cephalothin and ampicillin were also observed in bacteria isolated from dolphins, whales, and seals in the Northeastern United States Coast [35]. An overall high prevalence (88%) of resistance to at least one antibiotic was found among bacteria isolated from wild bottlenose dolphins in Florida, with highest resistances against erythromycin followed by ampicillin [6]. A previous study on antibiotic susceptibility patterns of bacteria isolated from stranded cetaceans in the Philippines reported the highest resistance (47%) to cefazolin [12]. Susceptibilities to amikacin and gentamicin were also reported among bacteria isolated from marine mammals in Florida, South Carolina, and Northeastern US Coast [5, 35].

Based on these findings, the choice of antibiotics for treating bacterial infections (most commonly pneumonia: pers comm., PMMSN veterinarians) caused by Enterobacteriaceae in locally stranded cetaceans under rehabilitation should consider the susceptibility and/or resistance profiles of bacteria. This may be possibly done through the stranding response being carried out by PMMSN, wherein such profiles can be provided by the collaborating microbiologists (e.g., the authors of this study) to the veterinarians handling the medical management of cetaceans. In the case of the bacteria isolated from cetaceans sampled in the present study, carbapenems are the most effective antibiotic. However, this information must be interpreted with caution, as the bacteria were not significantly associated with any clinical presentation of infection or disease in the cetacean.

The cetacean species sampled in this study generally inhabit deep waters, but their physiology entails a regular need to surface to sequester oxygen from the air for breathing, thus exposing themselves to sewage outflows and other forms of pollution that eventually reach them from the nearby coast [84–86]. The presence of bacteria (and associated antibiotic resistances) in these cetaceans indicate biological pollution and presence of antibiotic resistance in their habitats [87–89]. In this study, 33.33% of the isolates from cetaceans had MAR indices greater than 0.2, suggesting that the isolates may have developed resistance from sources that the cetaceans were exposed to, such as bodies of water highly polluted with antibiotics, including domestic, industrial and hospital sewage outflows, water-treatment facilities, and the like [85, 86]. As the use of antibiotics stems from anthropogenic activities, this implies the need to regulate and monitor the use and improper disposal of antibiotics to water bodies.

## Conclusion

Twenty-one cetaceans that stranded in different parts of the Philippines were sampled for bacterial isolation and antibiotic resistance screening as well as histopathological assessment of available tissues. In the absence of conclusive data on the specific causes of the mortality or morbidity of the cetaceans in relation to the stranding event, the histopathological findings just provide clues on possible involvement of factors (e.g., acoustic trauma, stress, etc.) that may have affected the health of cetaceans rendering them to strand or die, or possible effects of the stranding event itself on the animal. Bacteriological findings showed more than 50% of the isolated bacteria are multiple antibiotic resistant and that all of them are resistant to erythromycin and susceptible to imipenem, doripenem, ciprofloxacin, chloramphenicol, and gentamicin. While these information may be helpful in the medical management of stranded cetaceans during rehabilitation, they also indicate the extent of antimicrobial resistance in the marine environment. As sentinels, cetaceans demonstrate the threats faced by their populations in the wild, and monitoring their health through stranded representatives is a practical approach that can help improve conservation efforts. As local stranding network expands and veterinary and research expertise improve, more robust data from bacteriological and histopathological assessments of cetaceans are expected to be available in the coming years.
