# Correction: Bacteriological and histopathological findings in cetaceans that stranded in the Philippines from 2017 to 2018

**DOI:** 10.1371/journal.pone.0298904

**Published:** 2024-02-09

**Authors:** The PLOS One Staff

There are errors in the Funding section. The correct Funding statement is: MCMO and LVA received funding for the study through project grants: BIO-19-1-05 (UP-NSRI) and 202050 ORG (UP-OVCRD) for MCMO for the conduct of the methodology and 171704SOS (UP-OVCRD) for LVA for sample collection. The funders had no role in study design, data collection and analysis, decision to publish, or preparation of the manuscript.

The publisher apologizes for the error.
